# Fluoxetine Protects Retinal Ischemic Damage in Mice

**DOI:** 10.3390/pharmaceutics15051370

**Published:** 2023-04-29

**Authors:** Giovanni Luca Romano, Lucia Gozzo, Oriana Maria Maurel, Serena Di Martino, Valentina Riolo, Vincenzo Micale, Filippo Drago, Claudio Bucolo

**Affiliations:** 1 Department of Biomedical and Biotechnological Sciences, Section of Pharmacology, University of Catania, 95100 Catania, Italyluciagozzo86@icloud.com (L.G.);; 2 Center for Research in Ocular Pharmacology-CERFO, University of Catania, 95100 Catania, Italy

**Keywords:** neuroprotection, retinal ganglion cells, ischemia reperfusion, fluoxetine

## Abstract

Background: To evaluate the neuroprotective effect of the topical ocular administration of fluoxetine (FLX) in a mouse model of acute retinal damage. Methods: Ocular ischemia/reperfusion (I/R) injury in C57BL/6J mice was used to elicit retinal damage. Mice were divided into three groups: control group, I/R group, and I/R group treated with topical FLX. A pattern electroretinogram (PERG) was used as a sensitive measure of retinal ganglion cell (RGC) function. Finally, we analyzed the retinal mRNA expression of inflammatory markers (IL-6, TNF-α, Iba-1, IL-1β, and S100β) through Digital Droplet PCR. Results: PERG amplitude values were significantly (*p* < 0.05) higher in the I/R-FLX group compared to the I/R group, whereas PERG latency values were significantly (*p* < 0.05) reduced in I/R-FLX-treated mice compared to the I/R group. Retinal inflammatory markers increased significantly (*p* < 0.05) after I/R injury. FLX treatment was able to significantly (*p* < 0.05) attenuate the expression of inflammatory markers after I/R damage. Conclusions: Topical treatment with FLX was effective in counteracting the damage of RGCs and preserving retinal function. Moreover, FLX treatment attenuates the production of pro-inflammatory molecules elicited by retinal I/R damage. Further studies need to be performed to support the use of FLX as neuroprotective agent in retinal degenerative diseases.

## 1. Introduction

Glaucoma is a progressive optic neuropathy characterized by retinal ganglion cell (RGC) death, irreversible visual field loss, and high intraocular pressure (IOP) [1,2,3]. Sex hormones and aging have been suggested as driving the incidence of eye conditions such as glaucoma and ocular surface disease, among others [4].

Several causes of RGC damage have been proposed, including oxidative stress, mitochondrial impairment, axonal transport blockade, synaptic dysfunction, glutamate excitotoxicity, and changes in pro-inflammatory cytokines [5].

Pharmacological strategies targeting IOP (e.g., beta blockers, carbonic anhydrase inhibitors, prostaglandin derivatives, sympathomimetics, miotics, and Rho-Kinase inhibitors) have been demonstrated to slow down disease progression [6]. However, since glaucomatous damage persists in most patients despite IOP reduction, neuroprotective strategies to prevent or delay RGC loss have been investigated and demonstrated to be valuable treatment options [7]. Despite this, no preventive or curative treatments are available in clinical practice yet [8].

In this regard, second-generation antidepressants have demonstrated neuroprotective properties and have been investigated with regard to several neurodegenerative diseases [9,10,11,12,13,14,15,16,17]. In particular, treatment with selective serotonin reuptake inhibitors (SSRIs) has been associated with a reduced risk of developing Alzheimer’s disease (AD) and with the delayed progression of mild cognitive impairment (MCI), although it is unclear whether it can repair the damage once it is established [18,19,20,21]. Several clinical studies have reported an increased prevalence of glaucoma in patients with AD and dementia compared to control groups [22,23,24]. Bayer et al. (2002) showed a 26% prevalence of glaucoma in AD patients, compared to 5% in a control group [25,26]. Increased prevalence of cognitive impairment has likewise been shown to be associated with glaucoma patients [27].

Vision loss in retinal diseases occurs due to a variety of mechanisms including retinal ischemia [28,29,30]. General speaking, neurodegeneration is a common feature underlying irreversible vision loss in retinal diseases such as optic neuropathy [23,31,32]. RGCs provide the final loop between retinal processing and higher-order visual processing in the midbrain and cortex [33,34]. Thus, in glaucoma, even though the photoreceptors are healthy and able to detect light, the visual information travelling as electrical impulse is not transmitted to the midbrain for processing and interpretation [35]. Neurodegeneration, in terms of RGC death, is well-known in glaucoma patients [36]. The pharmacological treatment of glaucoma is basically aimed at maintaining IOP within a physiological range [37]. Anti-glaucoma medications target aqueous fluid production in the ciliary body and/or the outflow pathways (trabecular meshwork and uveoscleral) [38]. The drugs approved to decrease IOP do not have retinal protection effects, per se [39]. Therefore, there is a real unmet medical need in terms of retinal protection. RGCs play a crucial role in vision and their damage can lead to irreversible blindness, as seen in advanced glaucoma [5].

The pharmacological protection of these cells has been widely explored in previous decades as a potential approach to slow down the progression of retinal degeneration and maintain vision function. IOP elevations have been shown to elicit deep changes in RGC function and modify the expression of inflammatory biomarkers such as TNFα and IL-1β [40,41,42].

The underlying mechanism involved in SSRI neuroprotection has not been clarified yet. These drugs can exert their neuroprotective effect apart from the effect on serotonin by acting on inflammation, oxidative stress, and microglia activation [43,44,45,46,47,48,49,50,51,52,53,54]. Several in vivo studies have demonstrated that fluoxetine (FLX) can be used as a neuroprotective agent in different models of neuronal damage [16,44,55,56,57,58,59]. It has been demonstrated that FLX protects the brain after ischemia damage by decreasing early and long-term neuronal loss and inflammation, improving survival and functional recovery, and enhancing neurogenesis [14]. FLX has demonstrated important immunoregulatory and anti-inflammatory effects in neurodegenerative animal models, repairing the oxidative damage by reducing lipid peroxidation, suppressing the production of reactive oxygen and nitrogen species, and increasing the activity of antioxidant enzymes [56,57,60,61,62]. Moreover, protection from Aβ-induced damage has been demonstrated to be mediated by TGF-β1 [44].

The effects of serotonin (5-HT) on retinal physiology and physiopathology have been investigated using various experimental procedures. 5-HT has been found in different ocular tissues, and its receptor subtypes are expressed in the retina [63,64]. Retinal electrophysiological studies have demonstrated that the activation of 5-HT receptors support the processing of light signals [65]. Moreover, the role of the serotoninergic system has been investigated with respect to retinal neurodegenerative processes: e.g., the neuroprotective effect of the 5-HT_1A_R agonist was demonstrated in retinal degeneration models [66]. In the present study, we investigated the neuroprotective effect of FLX on a well-known in vivo model of retinal damage.

## 2. Materials and Methods

### 2.1. Materials

Fluoxetine was purchased from Merck (Milan, Italy), tiletamine + zolazepam (Zoletil^®^) from Virbac (Milan, Italy), and medetomidine (Dexdomitor^®^) from Vetequinol (Bertinoro, Italy). The oxybuprocaine (Novesina^®^) was procured from Laboratoires Thea, (Clermont-Ferrand, France). TRIZOL Reagent was obtained from Invitrogen, Life Technologies, (Carlsbad, CA, USA). All other compounds and reagents were purchased from Merck (Milan, Italy).

### 2.2. Animals

Male C57BL6/J mice (provided by Charles River Laboratories, Sant’Angelo Lodigiano, Italy) were kept in a room with a continuously controlled temperature and free access to food and water during a 12 h light/12 h dark cycle. All experimental procedures were carried out in accordance with the Principles for the Care and Use of Animals in Ophthalmic and Vision Research approved by the Association for Research in Vision and Ophthalmology (ARVO). The study was designed and carried out following the principles of the three Rs (replacement, reduction, and refinement) to ensure that use of animals was minimized, and that the welfare of animals was maximized. The study gained approval from the University of Catania (Italy) Ethics Committee (approval #343).

### 2.3. Retinal Ischemia

A validated retinal ischemia/reperfusion (I/R) model was used in the present study to establish the retinal injury [67,68,69,70]. This model consists of the induction of an acute ocular hypertension, which results in a rapid and progressive loss of RGCs [71,72]. Animals were anesthetized using tiletamine + zolazepam (60 mg/kg) and medetomidine (40 μg/kg) administered by intraperitoneal injection, combined with a topical instillation of 0.4% oxybuprocaine. During the experimental procedures, mice were placed on a heating pad to avoid hypothermia. A 32-gauge needle was introduced through the cornea into the anterior chamber of the left eye to increase the IOP (up to 90 mm Hg) using phosphate buffered saline (PBS) contained in a reservoir connected to the needle. The increase in IOP induced retinal ischemia, confirmed by the blanching of the anterior segment and the arteries of the eye. The needle was removed after 60 min of ischemia, to allow rapid reperfusion. A buffered, isosmotic, aqueous formulation of FLX (1%) was prepared. Ten microliters were instilled 60 min before ischemia and then 1 h and 2 h after reperfusion. This dose was chosen based on preliminary studies. The mice were divided into three experimental groups: control (CTRL, *n* = 6), ischemia/reperfusion (I/R, *n* = 6), and I/R + fluoxetine (I/R + FLX, *n* = 6). The animals were killed by cervical dislocation 72 h after I/R damage, then the eyes were enucleated and the retinas collected. RNA was extracted from the retina samples collected from mice using TRIZOL Reagent according to the manufacturer’s protocol. The A260/A280 ratio of the optical density of RNA samples was 1.95–2.01 (measured with a Nanodrop spectrophotometer ND-1000, Thermofisher). cDNA was synthesized from 500 ng of RNA with a reverse transcription kit (SuperScript™ IV Reverse Transcriptase ThermoFisher Scientific, Carlsbad, CA, USA).

### 2.4. Pattern Electroretinogram

We used the non-invasive pattern electroretinogram (PERG) analysis technique to assess the RGC activity and to evaluate the cell function in vivo [73]. This is a specialized electrophysiologic test used to measure the retinal function in response to a pattern stimulus. It represents a specific tool for detecting the onset and monitor the progression of retinal damage, including RGC dysfunction in animal models of glaucoma. Indeed, PERG analysis may provide important information to clarify the physiopatological mechanisms behind the glaucomatous disease, but also the effect of neuroprotective agents on retinal function. A common subcutaneous needle positioned on the snout with a commercially available instrument (Jorvec Corp., Miami, FL, USA) allowed for simultaneous recordings of the response from both eyes.

**Figure 1:**
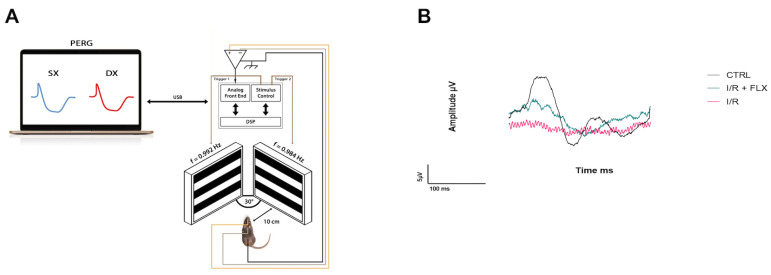
RGC function was assessed by the pattern electroretinogram (PERG). PERG amplitude values were significantly (*p* < 0.05) higher in the I/R FLX group compared to untreated I/R one, whereas the average PERG latency of I/R mice was significantly (*p* < 0.05) reduced compared to the control, suggesting the protection of RGC function. (**A**) Mouse PERG recording layout. (**B**) Representative waveforms of PERG of all experimental groups: control (CTRL), ischemia/reperfusion (I/R), and I/R + fluoxetine (I/R + FLX).

In particular, the recording electrode consisted of a thin silver wire of 0.25 mm diameter, configured into a semicircular loop with a 2 mm radius by bending the wire around a screwdriver with a 2 mm diameter. The two electrodes (one for the right eye and one for the left eye) had mirrored geometry. The vertical part connected to the electrode holder was located on the temporal side of the eye, so as not to interfere with vision. The electrodes were gently positioned on the corneal surface surrounding the undilated pupil through a precision device under microscopic control, without limiting the field of view. These procedures entailed minimal manipulation of the eye. Thanks to the thin diameter of the silver wire, small forward–backward movements of the eye associated with breathing were allowed, avoiding increased noise and corneal abrasions. This corneal stimulation can induce cataract in the animal, precluding further PERG recordings. The placement of the electrodes allowed us to verify the possible development of cataracts, as well as check that the pupil was focused on the pattern stimulus. Instillations of two microliters of balanced salt solution (BSS) were topically applied every 30 min to prevent corneal dryness, maintaining the cornea and lens in excellent condition throughout the recording. Anesthetized mice were transferred on a heating plate with their superior incisor teeth hooked to a bite bar and the head gently restrained by two ear knobs. The body was kept at a constant temperature of 37 °C using a feedback-controlled heating pad (TCAT-2LV, Physitemp Instruments, Inc., Clifton, NJ, USA). The mouse PERG recording layout is shown in Figure 1A. During the recording, visual stimuli (black and white horizontal bars generated on LED tablets) were presented at a 10 cm distance from each eye (56° vertical × 63° horizontal field; spatial frequency, 0.05 cycles/deg; 98% contrast; 800 cd/sqm mean luminance; left-eye reversal rate, 0.992 Hz; right-eye reversal rate, 0.984 Hz). The mean of the electrical signals was detected by the subcutaneous electrode inserted on the snout and calculated by the software (>1110 epochs), and PERG responses from each eye were isolated by averaging at a stimulus-specific synchrony. As previously reported, PERG waveforms include a positive wave (defined as P1) and a slower negative wave with a large depression (defined as N2). Subsequently, for each waveform, the peak-to-trough (P1-N2) amplitude (the PERG amplitude) and the time-to-peak of the P1 wave (the PERG latency) were measured [74].

### 2.5. ddPCR Analysis

For ddPCR technology (QX200 Droplet Digital PCR ddPCR™) System—Bio-Rad, (Hercules, CA, USA, 21 μL reaction mixtures containing 50 ng of cDNA, 250 nM of each forward and reverse primer, DNase free-water, and 11 µL of 2X QX200™ddPCR™ EvaGreen Supermix (cat. no. 1864034; Bio-Rad Laboratories, Inc.) were used. A measure of 20 μL of the reaction mix were obtained to generate droplets using the QX200 droplet generator according to the manufacturer’s instructions (Instruction Manual, QX200™ Droplet Generator—Bio-Rad). After generation, the droplets were transferred into a 96-well plate, sealed, and amplified in a T100 Thermal Cycler (Bio-Rad Laboratories, Inc.) under the following thermal conditions: enzyme activation at 95 °C for 5 min, 40 cycles of amplification at 95 °C for 30 s (denaturation) and 58 °C for 1 min (annealing/elongation), droplet stabilization at 90 °C for 5 min, followed by an infinite hold at 4 °C. A ramp rate of 2 °C/s was used throughout the steps. Following PCR, the plates were directly analyzed with a QX200 Droplet Reader—Bio-Rad to count positive and negative droplets. The data (copies/μL) were processed by using QuantaSoft version 1.7.4.0917 (Bio-Rad, USA) according to the Poisson distribution. A minimum of 11,000 acceptable droplets per 20 µL reaction were used. Each reaction was performed in duplicate, including also positive and negative controls. The sequences of all the primers (IL-6, TNF-α, Iba-1, IL-1β, S100β, HPRT) are listed in Table 1. All targets were previously assessed for reaction efficiency by a standard amplification curve generated by a real-time PCR reaction on 7900 HT. The absolute quantifications of all genes were normalized with the HPRT reference gene.

### 2.6. Statistical Analysis

The GraphPad Prism Software, version 8 (GraphPad Software, Inc., San Diego, CA, USA) was used to perform statistical analysis. PERG amplitude and latency, as well as the statistical significance of retinal mRNA expression between groups, were analyzed using one-way ANOVA followed by Tukey’s post hoc test for multiple comparisons. Student’s *t*-test was applied for single comparisons. To assess the data distribution, the Shapiro–Wilk normality test was carried out. The graphs are shown as columns (mean ± SD). We considered statistically significant *p* values ≤ 0.05.

## 3. Results

### 3.1. Retinal Function

**Figure 2:**
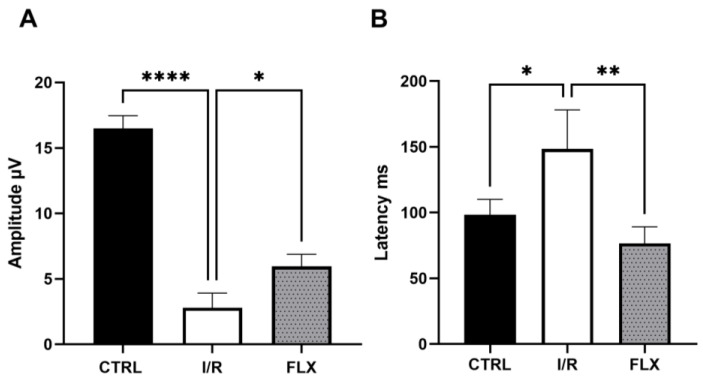
Comparison between PERG amplitude values (μV, (**A**)) and latency values (ms, (**B**)) in CTRL, I/R, and treated (FLX) mice. Fluoxetine counteracts RGC loss of function induced by I/R injury, after 72 h, in mouse retinas. In each panel, bars represent the mean values ±SD. One-way ANOVA analysis was performed, followed by the Tukey post hoc test. (**A**) * *p* < 0.05 I/R-FLX vs. I/R; **** *p* < 0.0001 I/R vs. CTRL; (**B**) ** *p* < 0.01 I/R-FLX vs. I/R; * *p* < 0.05 I/R vs. CTRL. *n* = 6.

Retinal function, measured with PERG, was reduced by more than 50% after I/R protocol, and this dysfunction was significantly (*p* < 0.05) attenuated by fluoxetine treatment (Figure 1 and Figure 2). Figure 1B shows representative waveforms recorded with PERG from each group. A comparison between the PERG amplitudes and PERG latency values recorded in the three groups (control group, I/R group, and I/R + fluoxetine group) was performed. As shown in Figure 2A,B, PERG amplitude values were significantly higher in the I/R FLX group compared to untreated I/R one (*p* < 0.05), whereas the average PERG latency was significantly reduced in I/R mice compared to the control animals (*p* < 0.05), suggesting the protection of RGC function.

### 3.2. Biomarkers

**Figure 3:**
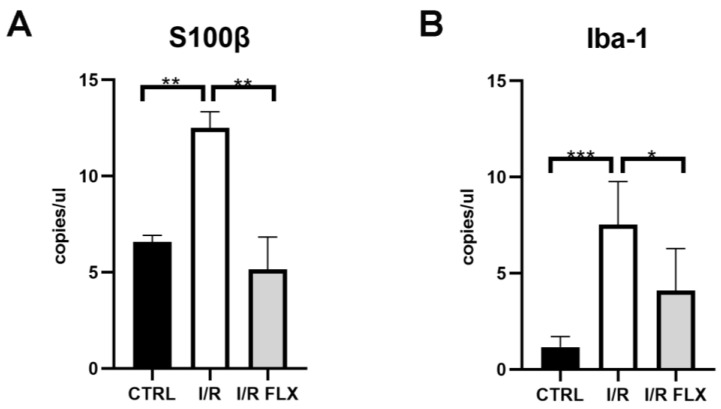
S100β and Iba-1 retinal mRNA expression in control mice (CTRL), ischemia/reperfusion injured (I/R) mice, and I/R mice treated with fluoxetine (I/R-FLX). S100β and Iba-1 were upregulated in I/R mice, after 72 h, compared to the CTRL group. Fluoxetine treatment counteracted the upregulation of S100β (**A**) and Iba-1 (**B**) elicited by I/R injury. The mRNA levels were evaluated by ddPCR; values are reported as mean values ±SD; *n* = 6. One-way ANOVA analysis was performed, followed by the Tukey post hoc test. Data are plotted as copies/μL. (**A**) ** *p* < 0.01 I/R-FLX vs. I/R, and I/R vs. CTRL; (**B**) *** *p* < 0.001 I/R vs. CTRL; * *p* < 0.05 I/R-FLX vs. I/R. *n* = 6.

**Figure 4:**
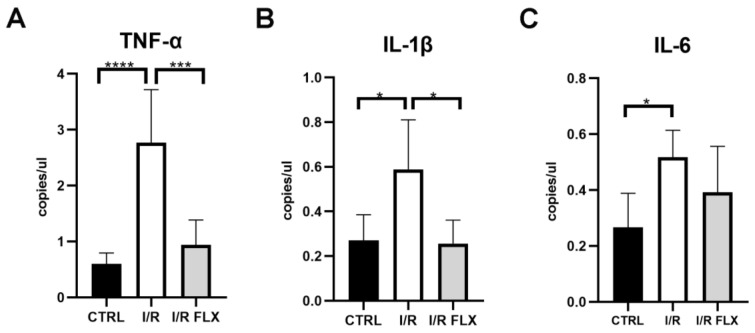
TNF-α, IL-1β, and IL-6 retinal mRNA expression in control mice (CTRL), ischemia/reperfusion injured (I/R) mice, and I/R mice treated with fluoxetine (I/R-FLX). TNF-α, IL-1β, and IL-6 were upregulated in I/R mice compared to the CTRL group. Fluoxetine treatment counteracted the upregulation of TNF-α (**A**) and IL-1β (**B**) elicited by I/R injury. The mRNA levels were evaluated by ddPCR; values are reported as mean values ± SD; *n* = 6. One-way ANOVA analysis was performed followed by the Tukey post hoc test. Data are plotted as copies/μL. (**A**) *** *p* < 0.0001 I/R-FLX vs. I/R, **** *p* < 0.001 I/R vs. CTRL; (**B**) * *p* < 0.05 I/R-FLX vs. I/R, and I/R vs. CTRL; (panel (**C**)) * *p* < 0.05 I/R vs. CTRL. *n* = 6.

In ocular neurodegenerative diseases, the involvement of retinal microglial activation as well as astrocytes and Müller glial cells is a common phenomenon; therefore, glia cell markers were assessed in the present study. As shown by the ddPCR results, I/R injury significantly (*p* < 0.05) upregulated the mRNA expression of several biomarkers in mice retina, whilst FLX was able to significantly (*p* < 0.05) attenuate them (Figure 3; Figure 4). S100β and Iba-1 expression were significantly (*p* < 0.05) increased in mice retina after I/R injury, and FLX significantly (*p* < 0.05) counteracted the expression (Figure 3). We also assessed different pro-inflammatory molecules, such as TNF-α, IL1β and IL-6, to figure out the effect of fluoxetine on mice retina after I/R injury (Figure 4). The results showed retinal overexpression of these biomarkers after I/R injury, which was significantly (*p* < 0.05) counteracted by fluoxetine treatment, except for IL-6.

## 4. Discussion

Glaucoma still represents an important unmet medical need due to the lack of treatments able to prevent RGC loss and restore retinal function. RGC death in glaucoma can be related to several cytotoxic factors, including deprivation of neurotrophins, glutamate excitotoxicity, mitochondrial dysfunction, ischemia, and oxidative stress [75,76]. It is well known that oxidative stress and neuroinflammation are among the main causes of several neurodegenerative disorders [77].

The retinal ischemia-reperfusion injury model mimics clinical situations and is recognized as a well-known animal model for studying RGC dysfunction after ischemic damage. The injury of the ischemic tissues is determined not only by the lack of blood supply, but also by the process of reperfusion itself by the generation of free radicals and inflammatory cytokines. The shortage of oxygen and nutrients resulting from I/R injury generates reactive oxygen species, leading to retinal damage and the expression of several pro-inflammatory molecules [78,79,80,81,82]. As for other neurodegenerative diseases, neuroinflammation, elicited by ischemia, represents a key process in glaucoma physiopathology [83]. Besides RGCs, other cell types are involved in retinal diseases, including reactive glial cells, which induce prolonged inflammation that can contribute to retinal cell death, axon degeneration, and the loss of synapses [83,84,85,86,87,88,89].

Neuroinflammation represents one of the main contributors to RGC loss in glaucoma [90], similarly to other neurodegenerative disorders, including amyotrophic lateral sclerosis (ALS), AD, Parkinson’s disease (PD), Huntington’s disease, and frontotemporal dementia.

A considerable amount of evidence recognizes TNFα as one of the most important players in glaucoma, even though its exact role in the disease progression remains unclear [91]. Since it was first discovered as a cytotoxic agent for tumor cells, TNF is known as a potent mediator in the apoptotic processes in general and as regulator of the immune response, [92,93] and has been demonstrated to be involved in the pathogenesis of human diseases. It is the dominant mediator in pro-inflammatory processes, put in place to protect the CNS in response to deleterious stimuli, but may become the key neuroinflammatory mediator of neurotoxicity and neurodegeneration, inducing microglial activation, synapse loss, and the propagation of the inflammatory state [94,95].

In response to injuries, proinflammatory cytokines, such as TNF-α, are released by endothelial cells, neurons, glial cells, and infiltrating immune cells, leading to the activation of the MAPK cascade [96,97]. The finale effect is the activation of microglia, astrocytes, and the NF-κB pathway and increased proinflammatory gene expression [98,99,100].

Growing evidence supports the role of TNF-α as a mediator of RGC death in glaucoma through the binding of TNF receptor-1 (TNF-R1) [101]. Previous studies have shown increases in the glial production of TNF-α, and the upregulation of TNF-R1 in RGCs in the eyes of patients with glaucoma [102,103]. Moreover, TNF can act through TNF receptor-2 (TNF-R2), which is minimally expressed physiologically in the CNS but is upregulated in neurological diseases. The TNF pathway has also been associated with the activation of survival signals mediated by specific receptors, exemplified by differential responses of RGCs and glial cells [101,104,105]. In this regard, Fontaine et al. assessed the role of TNF receptors in preclinical models deficient for TNF-R1, TNF-R2, or TNF, quantifying neuronal cell loss in the ischemia reperfusion-induced retinal damage [104]. This study showed no effect of TNF deficiency on the overall cell loss, whereas the absence of TNF-R1 or TNF-R2 led to opposite results: namely, the reduction and the enhancement of neurodegeneration, respectively. These results demonstrate the different actions of TNF in the retina mediated by TNF-R1 and TNF-R2 in promoting neurodegeneration and neuroprotection.

Certainly, a better understanding of the role of TNF may allow us to the develop better treatments for glaucoma in terms of retinal protection [91,106]. Therefore, the selective inhibition of TNF-R1 may represent a strategic pharmacological approach to reducing retinal damage, potentially superior to the suppression of TNF activity in general [104].

The above-mentioned pathways represent a potential target for neuroprotective molecules, which can be a valuable strategy to preserve RGCs, especially if administered in the early phases of cell dysfunction preceding death [71,74,107,108,109].

Several options have been explored and demonstrated to prevent or delay visual impairment and RGC loss [110,111], but need further investigation to be introduced in clinical practice. The administration of neurotrophins, such as brain-derived neurotrophic factor (BDNF), into the eye have been demonstrated to increase the survival of RGCs and to preserve retinal function [112].

In the present study, we showed that topical treatment with fluoxetine protected RGC function from I/R damage and reverted the increase in several inflammatory markers, as well as activating glial cells. The data generated in the present study are in accordance with other evidence on brain ischemia in animal stroke models, showing the efficacy of fluoxetine as a neuroprotective, anti-inflammatory, and neurorestorative drug [14].

Relevant non-clinical studies have demonstrated the neuroprotective properties of fluoxetine, even though the underlying mechanisms have not been clarified yet [113].

Fluoxetine is a classical selective SSRI widely used in the treatment of depression [114]. However, apart from its effects on serotonin, the drug has also demonstrated anti-inflammatory, anti-tumor, and neuroprotective effects [9]. Preclinical and clinical evidence has identified inflammation as a key factor in the physiopathology of depression [115]. Inflammation induces depressive-like behaviors in animal models, such as anhedonia; decreases in exploratory, social behaviors, and food intake; and sleep disorders [116]. This response is considered an adaptive mechanism which contributes to the fight against infections and accelerates healing. However, prolonged inflammation maintained by ongoing stress can be harmful, increasing the risk of depression and other diseases [117].

It has been demonstrated that this drug can modulate pro-inflammatory cytokines, including IL-1β, IL-6, and TNF-α. The evaluation of 292 individuals with major depression treated with fluoxetine for at least 6 weeks showed decreased levels of IL-1β, IL-6, or TNF-α at the end of the antidepressant treatment [118].

Moreover, fluoxetine is able to act on neuronal injury induced by ischemia/reperfusion damage and neuronal apoptosis induced by inflammation, mitigating neurological deficits [119]. Thus, in addition to the neurotransmitter hypothesis, fluoxetine can suppress neuronal death associated with chronic stress and depression and induced by neuro-inflammation [113].

The identification of the molecular actors involved in this process would improve knowledge regarding the therapeutic effects of SSRIs in depressive disorders, but also their potential in the treatment of neurodegenerative diseases.

The neuroprotective effects of fluoxetine are not clear, and several mechanisms of action were proposed. It has been hypothesized that the neuroprotective effects of SSRIs are linked to their immune-regulatory properties and to a reduction in reactive oxygen and nitrogen species, also thanks to the stimulation of the antioxidant defense [45,49]. Indeed, fluoxetine has been demonstrated to reduce the lipid peroxidation and increase the activity of antioxidant enzymes in animal models [60,61]. In vitro studies found that glial cells were essential to mediate the neuroprotective effects of SSRIs, including fluoxetine, being active in mixed neuronal cultures but not in pure neuronal cultures [120,121]. Fluoxetine given by intraperitoneal injection in a mouse model of optic nerve crush (ONC) resulted in a reduced number of microglia/macrophages at the lesion site compared to the control [122].

We hypothesized that the retinal protective effect of fluoxetine is related to attenuation of neuroinflammation through different pathways such as cytokines signaling as showed in the present study.

The ionized calcium binding adaptor molecule 1 (Iba1) is a protein that takes part in the process of phagocytosis and is considered a marker of microglia activation in several neurological damages. Iba1 expression has been demonstrated to be correlated with microglial activation in retinal injury [123]. In accordance with these findings, we observed the upregulation of Iba1 resulting from I/R damage, reverted by fluoxetine treatment. Furthermore, the S100B glial marker, overexpressed after retinal ischemia, was attenuated by fluoxetine treatment. Finally, we showed a significant attenuation of inflammatory molecules by fluoxetine treatment after retinal I/R insult. The function of the retina, assessed by PERG, was preserved by fluoxetine treatment.

## 5. Conclusions

In conclusion, we demonstrated that ocular topical treatment using fluoxetine protects retinal tissue after ischemia, attenuating the expression of inflammatory molecules, and preserving retinal function. The findings open the way to further investigate the interesting molecule fluoxetine both in vitro and in vivo. Finally, these data warrant further clinical evaluation of fluoxetine for the treatment of glaucoma.

## Figures and Tables

**Table 1:** PCR primer sequences.

Gene	Forward Primer	Reverse Primer
IL-6	5′-ACAACCACGGCCTTCCCTA-3′	5′-TTGCCATTGCACAACTCTTTTCTC-3′
TNF-α	5′-CAGGCGGTGCCTATGTCTC-3′	5′-CCATTTGGGAACTTCTCATCCCTT-3′
Iba-1	5′-GCAATTCCTCGATGATCCCAAA-3′	5′-GATCAAACTCCATGTACTTCACCTT-3′
IL-1β	5′-GGGCCTCAAAGGAAAGAATC-3′	5′-TACCAGTTGGGGAACTCTGC-3′
S100β	5′-ACTTCCTGGAGGAAATCAAGGAGC-3′	5′-ACACTCCCCATCCCCATCTTC-3′
HPRT	5′-TCAGTCAACGGGGGACATAAA-3′	5′-GGGGCTGTACTGCTTAACCAG-3′

## Data Availability

The raw data supporting the conclusion of this article will be made available by the authors, without undue reservation.
